# Barriers for conducting clinical trials in developing countries- a systematic review

**DOI:** 10.1186/s12939-018-0748-6

**Published:** 2018-03-22

**Authors:** Chalachew Alemayehu, Geoffrey Mitchell, Jane Nikles

**Affiliations:** 1 Faculty of Medicine University of Queensland, Brisbane, Australia; 2 The University of Queensland Centre for Clinical Reseaerch (UQCCR), Brisbane, Australia

**Keywords:** Barriers, Clinical trials, Developing countries

## Abstract

**Background:**

Clinical trials for identification of efficient and effective new diagnostic and treatment modalities are needed to address disproportionately high burden of communicable (e.g., HIV/AIDS, tuberculosis, and malaria) and non-communicable diseases (e.g., diabetes) in developing countries. However, gross under-representation in global clinical trial platforms contributes to sustained health inequity in these countries. We reviewed the literature on barriers facing clinical researchers in developing countries for conducting clinical trials in their countries.

**Methods:**

Literature indexed in PubMed, Embase, CINAHL and Web of Science, WHO Global Health Library were searched. Grey literature was also searched. Search key words included barriers, challenges, clinical trials and developing countries. Articles within the scope of this review were appraised by two reviewers.

**Results:**

Ten studies, which are reported in 15 papers, were included in this review. Following critical review we identified five unifying themes for barriers. Barriers for conducting clinical trials included lack of financial and human capacity, ethical and regulatory system obstacles, lack of research environment, operational barriers and competing demands.

**Conclusion and recommendation:**

There were substantial barriers at system, organization and individual level. We propose that to address this problem, instituting a system for wider implementation of local investigator-initiated trials is warranted. These trials are more applicable to local populations because they build on local healthcare knowledge. They are more demand-led, influence policy and responsive to a country’s needs because they are driven by a local or national agenda.

**Electronic supplementary material:**

The online version of this article (10.1186/s12939-018-0748-6) contains supplementary material, which is available to authorized users.

## Introduction

Developing countries represent the majority of the world’s population [1]. These countries host nearly 90% of the worldwide burden of disease, most of which comprises preventable infectious diseases [2]. There is also an increasing prevalence of non-communicable diseases (NCDs). The transition imposes a double burden [3] on the already pressured resources in developing countries.

These countries’ health care systems need evidence to guide decisions about the most efficient and cost-effective interventions for them. The shortage of resources in developing countries paradoxically increases the need for reliable healthcare evidence to prioritize the use of their scarce resources [4]. According to the World Bank, most developing countries have a Gross National Income per capita of under US$4036 [5]. Although developing countries bear the greatest burden of disease in the world, substantial research and development activity to address this inequity is lacking. These countries are under-represented in research due to lack of commercial viability and research capacity [6, 7], yet it is in these poorest regions where research-led solutions could bring the greatest impact to high rates of early mortality [8, 9].

Moreover, addressing context-specific questions is fundamental to designing interventions that improve health outcomes [10]. Medical treatments need to reflect biological and non-biological variations that exist across the world. Clinical trial research, particularly systematic studies in human subjects (including patients and other volunteers) are required in order to discover or verify the effects of and/or identify any adverse reaction to investigational products in diverse populations [11]. There are great differences in cultures and perceptions across the globe, and what is appropriate in one place might not be in another [12]. For example, some interventions shown to be efficacious in high-income countries are not similarly effective when used in other contexts [13, 14].

At the same time that the priorities of developed countries drives the research agenda of pharmaceutical companies, there is a disturbing underrepresentation of research addressing priority issues for developing countries [15, 16]. Diseases of relevance to high-income countries are investigated in clinical trials seven to eight times more often than diseases whose burden lies mainly in low-income and middle-income countries [17]. More than 80% of clinical trials listed on clinicaltrials.gov [18] are conducted in the developed world.

In addition, some of the trials being conducted in developing countries are seek to answer the questions of the developed world. A recent review indicated that about one-third of 509 clinical trials sponsored by US-based companies from 1995 to 2005 were conducted outside the USA, many in poor and low-income countries [19], without targeting diseases prevalent in these countries. Another study also found that only 10 of 1556 new drugs produced between 1975 and 2004 were targeting diseases specifically prevalent in poor and low-income countries [20].

There is also a growing realisation that many countries in the developing world, have not been exploiting the enormous research potential offered by their health care services [21], to answer research questions of the developed world. This potential includes reduced cost and time to recruit patients [9, 22] and increased incidence of diseases (eg cardiovascular diseases, diabetes, cancer) [23] of interest to the developed world.

A systematic review of barriers for conducting clinical trials by clinicians in developed countries identified several major barriers. These include time constraints, lack of staff and training, worry about the impact on the doctor-patient relationship, concern for patients, loss of professional autonomy, difficulty with the consent procedure, lack of rewards and recognition, and an insufficiently interesting question [24]. Barriers for conducting clinical trial vary between countries, and barriers to conducting clinical trials not normally considered by research institutes (local structural, infrastructural, and procedural aspects) may affect investigators more in poorer settings than in developed countries [25]. Even within the paucity of literature on conducting clinical trials in low resource countries, the literature mostly relates to ethical issues [19, 26–28].

Recently there have been calls from within the developing countries for more ownership over priority setting and research conducted in line with national health strategies [8, 29]. If enhancing clinical trials in developing settings is being considered, then identifying barriers and designing context-appropriate strategies are essential.

The objective of this study is to conduct a systematic review that assesses barriers for conducting clinical trials in in developing countries.

This review will enable entities contemplating clinical research in these countries to prepare and plan ahead, to minimize the impact of barriers, and thus contribute to a greater proportion of the world’s trials being conducted where the majority of people reside. Conducting more clinical trials in these environments will build confidence in the ability to perform them well, and many under-resourced people will benefit in the long term.

## Methods

**Fig. 1:**
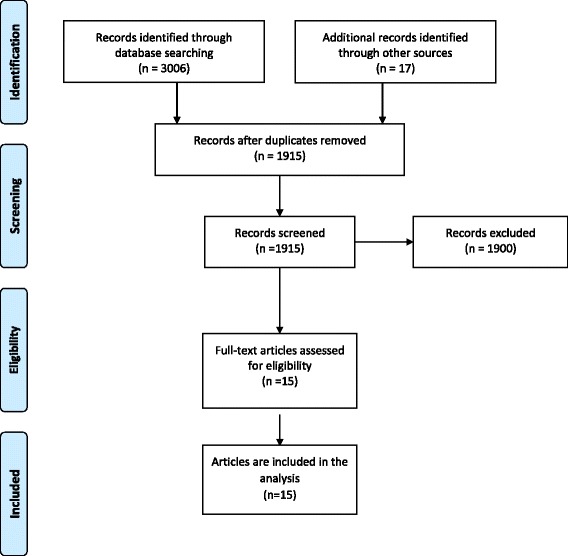
PRISMA 2009 flow diagram for study selection

This review is prepared based on the Preferred Reporting Items for Systematic Reviews and Meta- Analyses (PRISMA) statement checklist and flow diagram [30], modified for health service reviews [31]. The PRISMA checklist can be found in Additional file 1. A PRISMA flow chart outlined the study selection process [30] (see Fig. 1).

### Eligibility criteria

Included studies must:

1. Report on barriers/challenges for conducting research, specifically clinical trials within developing countries
2. Be published empirical, peer reviewed research studies (commentaries, editorials and literature reviews were excluded).
3. Published in English.
4. Bublished between 1995 and October 2015.

The Commission on Health Research for Development is an international initiative to study the status, effect, and needs of research on the health problems of developing countries. In the early 1990’s, the commission stated that the rapid expansion of country-specific health research to meet health needs of these countries was a necessity to encourage better health status for more people in settings with sometimes severely limited resources [32]. In 1995, WHO published Guidelines for Good Clinical Practice [11], the world’s first standard for reporting clinical trials. We selected this as a logical starting point for assessing clinical trials from the modern era. In 2015, a review findings regarding the distribution of global clinical trials was released [33]. This report concluded that despite the overall increase in clinical trials over the last two decades, the progress of clinical trials in developing countries was particularly slow and challenging. This milestone was accepted as a good point to conclude the search.

### Search strategy

The search strategy aimed to find both published and unpublished studies. A three-step search strategy was utilized. An initial limited search of MEDLINE was undertaken followed by analysis of the text words contained in the title and abstract, and of the index terms used to describe the article. A second search using all identified keywords and index terms was undertaken across all included databases, including a range of terms such as barriers, challenges, clinical trials and developing countries. Thirdly, the reference list of all identified reports and articles was searched for additional studies. To maximize sensitivity, a broad search strategy without country economic context was also used.

The databases searched included: PubMed, Embase, CINAHL, Web of Science, and WHO Global Health Library. Grey literature was identified by searching through the Network Digital Library of Theses and Dissertations, ProQuest Dissertations and Theses, and Google Scholar.

For example the PubMed search was:

((((Barriers [Title/Abstract]) OR Challenges [Title/Abstract])) AND ((clinical trials [MeSH Terms]) OR clinical trials [Title/Abstract])) AND ((((developing countries [Title/Abstract]) OR Third world countries [Title/Abstract]) OR Low income countries [Title/Abstract]) OR Middle income countries [Title/Abstract]).

Titles and abstracts of all the retrieved bibliographic records were screened two independent reviewers (CMA, JN). Full texts of potentially eligible records passing the title and abstract screening were retrieved and examined according to the eligibility criteria.

### Assessment of methodological quality

Eligible papers were assessed by two independent reviewers (CMA, JN) for methodological validity prior to inclusion in the review. Quality of each selected quantitative study was assessed using criteria adapted from the Newcastle-Ottawa scale for cross-sectional studies [34]. The scale gives a maximum of eight stars to each study: five stars for the representativeness of the sample: three stars for the adequate ascertainment of the outcome. We defined studies of high quality as those that scored the maximum 7 or 8 stars on the Newcastle-Ottawa scale; studies of medium quality scored 5 or 6 stars.

Critical appraisal of qualitative studies was made using criteria proposed by Kuper [35]. The criteria contains six major questions that address different key areas (design, sample, data collection, analysis, reporting), each of which are answered as ‘good’, ‘fair’ and ‘poor’. Based on literature [36], a quality score for each question was given using the following definitions: Good: Sufficient details/information are provided and well justified. Fair: most information is given and justified but some are missing. Poor: No or few descriptive details are provided.

The purpose of the quality assessment was not to establish thresholds below which studies were excluded, rather to distinguish between studies in terms of overall contribution to the review [36]. Any disagreements that arose between the reviewers were resolved through discussion, or with a third reviewer (GM).

### Data extraction and synthesis

We carried out the synthesis in two stages: a general mapping which described the characteristics and key findings of individual studies; and an in-depth review synthesizing the findings across all studies. Evidence was synthesized using a thematic analysis/synthesis [37], where important or recurrent themes were identified by tabulating key information across studies. To avoid potential restrictions (from using predetermined themes) and to allow the possibility of including emergent key concepts, we used the study findings themselves to conduct a thematic analysis.

First two reviewers (CM and JN) independently identified key concepts, then compared and analyzed them to identify predominant/recurrent themes across studies. The process of finding key concepts in each study was not always straightforward in the qualitative studies. This was particularly difficult in studies that made relatively simple analyses of their findings by describing what their participants reported, without reporting further analysis that identified key findings. As this process was dependent on the judgement of the reviewers, we repeated the process as a group. Through the group discussion, abstracted key concepts and subsequent themes were examined and changes made as necessary. This process was repeated until consensus was reached regarding the sufficiency and appropriateness of the themes and subthemes developed to explain barriers for conducting clinical trials in developing countries.

The synthesis approach allows significant findings of the review question to be summarized under thematic and sub thematic headings. Meta syntheses offer novel interpretations of findings [38]. However, due to the widely diverse settings as well as variations in methodological approaches across studies (which are important considerations in developing a more generalized model [38, 39]), we did not conduct a meta-synthesis. Finally, as the key findings were similar across studies, we chose to present and discuss the unifying themes and include both for the quantitative and qualitative studies in each theme.

## Results

The Embase search returned the highest number of publications (1076). Web of Science, PubMed, Cinhal and WHO Global Health Library returned 760, 712, 267 and 191 publications respectively. Additional applicable papers were included using the reference lists of these publications. After removing duplicates, the topic and abstracts of 1915 published articles were screened to check if they were eligible for this review. 1900 articles were excluded as they were out of the scope of this review (see Fig. 1), leaving ten studies reported in fifteen papers to be analysed [40–53].

### Selected studies

Ten studies, which were reported in 15 eligible papers, included in this review. One study [41] included seven countries (four from low and middle income countries (LMIC) and three from high income countries (HIC)), with a separate publication of the responses from each of the representatives of included countries. Therefore, we included the four papers from LMIC [42–45]. Two of the studies included in this review [40, 41] were global studies. Though these studies had a global focus, they assessed and clearly presented their results regarding barriers and facilitators of clinical trials in developing countries. (See Table 1).

**Table 1:** Summary of included studies

Author, location	Subjects (n)	Type	Focus	Main findings
Seruga, Africa: 4 countries, Asia: 5 countries, Central/ South America: six countries and 8 developed countries [40]	Oncologists with clinical trial experience (39 from LMICs and 41 from HICs)	Quantitative/Web based survey	Barriers	Lack of funding, lack of competent authorities and ethical procedures, lack of research materials, lack of time or competing priorities, concerns about insurance/indemnification coverage, lack of trained personnel, lack of patients/patient accrual
Schlaff, Latin America (Chile), the Middle East (Egypt), Europe (Greece), China, India, Australia, and the, United States [41–45]	Senior and accomplished investigators (7 participants, one from each country)	Qualitative	Barriers	India: Funding is limited, regulation impediments, misinformation about research Egypt: lack of funding, lack of a scientific atmosphere, and “brain drain” of scientists China: Combine the clinical with basic research Latin America: Less value for scientific research, insufficient allocation of human and economic resources.
Siegfried, South Africa [46]	Investigators and other clinical stakeholders (19)	Qualitative/ Key informant interviews	Barriers and need for additional training	Impediments in ethics processes, high cost of trials, the potential burden of trial conduct on clinical care, insufficient skilled researchers
Ranasinghe, China [47]	Health professionals conducted (40)	Qualitative/in-depth interviews	Barriers	Lack of leadership support for implementing quality improvement, lack of resources, fears of patient disputes and litigation, healthcare funding constraints, high out-of-pocket expenses, and patients’ refusal to participate
Franzen, Ethiopia [48]	Health researchers and stakeholders with research interest (20)	Qualitative/ in-depth interviews (*n* = 7) and focus group discussions (*n* = 3).	Barriers and facilitators	Barriers: Human and material capacity, regulatory and other administrative bottlenecks, operational hurdles, awareness, confidence and motivation
Franzen, Ethiopia and Cameroon [49]	Local health-researchers, senior stakeholders and regulators (72)	Qualitative/ in-depth interviews (*n* = 22), focus group discussions (*n* = 9), and process mapping exercises (*n* = 7)	Barriers and facilitators	Shared barriers: System and organisational barriers like low resources, weak regulatory and administrative systems, few learning opportunities, little human and material capacity, and few incentives for doing research. Ethiopia: lack of awareness, confidence, and motivation to undertake trials Cameroon: environments that discourage personal initiative were more problematic.
Cardoso, Sub-Saharan Africa (*n* = 46) [50]	303 key-informant interviews (clinical researchers and other clinical trial stakeholders)	Qualitative/key informant interview and literature review	Status and barriers	Levels/sustainability of funding, policymakers’ understanding of the importance of research, infrastructure in research institutions and Human resources available.
Gomez, Latin America countries (*n* = 15) [51]	92 medical oncologists	Quantitative/Survey	Barriers	Complex regulatory process, low budgets, high costs, poor financial management, and time constraint.
Sulthan, Saudi Arabia [52]	100 clinical researchers	Quantitative/Survey	Perception and barriers	Long approval process, shortage of human resource and lack of the institutional support, lack of encouragement, lack of awareness among the research professionals and the general public
Al-Dorzi, Saudi Arabia [53]	186 medical staff	Quantitative/Survey	Interest and barriers	lack of time, financial compensation and encouragement and lack of training of research

Regarding health conditions, six (60%) of included studies assessed barriers of conducting clinical trials on non-specific health conditions. The remaining four studies assessed barriers to conducting clinical trials in cancer [40, 51], reproductive disease [41] and acute coronary syndrome [47]. Of ten articles included studies, six used qualitative methods [41, 46–50] such as focus group discussion and individual interviews, and four articles [40, 51–53] used quantitative methods.

### Quality of included studies

We assessed the quality of all included studies and incorporated studies in the review. We believed that, given the paucity of literature on the topic, an inclusive approach would increase the collection of the diverse perspectives and experiences in a largely unexplored area.

Of the four quantitative studies, one study was high quality, two were medium quality and one was low quality. Of the medium quality studies, one had a low response rate (92 out of 404–23%) [51]. In the other study, sample size was not justified and there is no description of the sampling strategy [52]. The study considered to be of low quality showed a high risk of selection bias secondary to purposive sampling procedure for participant selection as well as a low response rate (27%). Moreover, a comparison between respondents and non-respondents was not provided as demographic data were obtained only for the respondents [40]-see Additional file 2: Table S1.

Most of the included studies [41, 46–50] relied on qualitative methods. For the majority of questions, all except one study [41] scored “good” for most criteria. The proposal [41] which led to publication of participants’ views from each represented country [42–45] did not present some important aspects of qualitative studies: the specific qualitative approach was not described and the research report did not describe the process of data collection, analysis and interpretation- Additional file 3: Table S2.

### Barriers to conducting clinical trials

Barriers to conducting clinical trials in developing countries were: lack of financial and human capacity, ethical and regulatory system obstacles, lack of research environment, operational barriers and competing demands (see Table 2).

**Table 2:** Thematic and sub thematic presentation of barriers for conducting clinical trials

No	Barriers for conducting clinical trial		
	Thematic barriers	Sub-themes	References
1	Lack of financial and human capacity	Lack of funding	[40–51]
		Lack of skilled personnel	[40, 42, 44, 45, 48–50, 52, 53]
		Lack of awareness and motivation	[43, 45, 48, 49, 52, 53]
2	Ethical and regulatory system obstacles	Delay of approval decisions	[40, 42, 45, 46, 48, 49, 52, 53]
		Unskilled authorities	[42, 45, 48, 49]
		Complex and strict ethical and regulatory system	[48, 49, 51].
4	Lack of research environment	Lack of infrastructure	[45, 47–50]
		Lack of research materials/facilities	[40, 46, 47]
		Lack of conducive scientific atmosphere	[43, 45, 50]
5	Operational barriers	Unsupportive administrative system	[43, 45, 47, 48, 51, 52]
		Lack of/difficult patient recruitment	[40, 44, 47]
6	Competing demands	Lack of time	[40, 48, 51, 53]
		Other competing priorities	[40, 43, 45, 46, 48, 51, 53]

#### Lack of financial and human capacity

Financial and human capital barriers were reported across all articles. Limited capacity in terms of funding were reported in all except two [52, 53] studies conducted in Saudi Arabia [52, 53]. Regarding human capacity, both lack of skilled personnel [40, 42, 44, 45, 48–50, 52, 53] and lack of awareness and of motivation to participate [43, 45, 48, 49, 52, 53] were reported as barriers.

#### Ethical and regulatory system obstacles

Delays in regulatory and ethical review were mentioned in most articles [40, 42, 45, 46, 48, 49, 52]. The time from initiation of all regulatory procedures to the actual start of the trial was very long. Describing the exact period was not common but one study stated that it was not uncommon for grants to expire before all approvals were in place. Four articles reported that the presence of unskilled authorities in the review process posed a barrier to conducting clinical trials [42, 45, 48, 49]. Over-complex and unreasonably strict ethical and regulatory systems were also reported in some articles [48, 49, 51].

#### Lack of research environment

Absence of suitable research infrastructure was stated by many articles [45, 47–50]. Lack of research materials for conducting clinical trials [40, 46, 47] and lack of conducive scientific atmosphere (including policy) [43, 45, 50] were also reported. One article reported lack of policymakers’ understanding of the importance of research as barrier [50].

#### Operational barriers

The majority of the articles [43, 45, 47, 48, 51, 52] stated that the administrative environment was characterized by lengthy and complex logistic and financial systems that hampered the conduct of clinical trials. Some articles reported difficulty in patient recruitment [40, 44, 47] as a barrier for conducting clinical trials.

#### Competing demands

Many articles reported that lack of time [40, 48, 51, 53] and other competing priorities related to the competing demands of working multiple jobs and their attendant responsibilities [40, 43, 45, 46, 48, 51, 53] was an issue. Most senior staff for conducting clinical trials were clinicians or academic staff whose primary jobs still had to be prioritised.

### Comparison of barriers by stakeholders

Two major stakeholder groups were identified from the included studies: researchers and health professionals. Four [40, 47, 51, 53] of the ten included studies reported findings from health professionals, the remaining six studies involved health researchers [41–46, 48–50, 52]. We compared themes and subthemes across these groups to identify similarities and differences. With the exception of concerns around time [40, 48, 51, 53], reported barriers for conducting clinical trials were similar between these two groups, with financial and human barriers being the most common in both groups. There were, however, a considerable variation regarding lack of time as a barrier, with most (3 out of 4) studies that reported views of health professionals [40, 51, 53] putting more emphasis on lack of time and only one study [48] that involved clinical researchers reporting lack of time as a barrier.

## Discussion

Although clinical trials are important to address sustained inequity that results from high burden of disease in developing countries, these countries are grossly under-represented in global clinical trial platforms. Currently, less than 20% of clinical trials are being conducted in developing countries [54], and only 1% of the recently discovered drugs are aimed at management of tropical diseases [55].

We reviewed the literature on barriers facing clinical researchers in resource-poor settings, and found only a small number. Clinical researchers conducting clinical trials in low resource settings faced a range of substantial barriers at all levels, starting from the system level, to the institute level, to the individual level. The greatest challenge that faced researchers in developing countries was lack of financial and human capacity. In addition, several other themes emerged from the research literature: ethical and regulatory system obstacles, lack of research environment, operational barriers, and competing demands.

By contrast, a systematic review related to physician barriers in RCTs for cancer and other illnesses in the developed world identified lack of time as a major barrier [24]. Another systematic review identified barriers as system-organization barriers (time involvement and resource issues), t*rial design-related barriers*, and physician-related barriers [56].

Although many of the issues confronting clinical trialists working in resource-limited settings are the similar, the human and other resource capacity of developing countries lags far behind that available in wealthier nations [57]. Based on their experience, several authors who have worked as a clinical investigator in developing countries have published their perspectives regarding challenges for conducting clinical trials. Ethical and regulatory issues, administrative issues, lack of finance, lack of infrastructure, poor data quality, and lack of training curricula focusing on clinical research were the major bottlenecks [58, 59]. One article discussed the regulatory challenges associated with conducting multi-country clinical trials in resource-limited settings (Africa, Asia, South America, and the Caribbean) [60]. The authors reported that the regulatory processes in resource limited countries hinder the efficient implementation of multi-site clinical trials, delaying research important to the health of populations in these countries and costing millions of dollars a year.

Lack of funds was the most commonly cited reason reported in the included studies. This is reflective of the 10/90 gap, in which less than 10% of health research funds in the world are directed toward problems that affect 90% of the world’s population, and an even smaller percentage go to fund researchers and health problems indigenous to developing countries [6, 7, 61]. Funding for clinical trials in developing countries comes mostly from Western countries and the pharmaceutical companies based there [62]. In most low-income countries, research is a luxury because of economic constraints [63]. Scarce resources in developing countries are nearly all spent on program implementation, and allocating funds for research is almost out of the equation in most development plans. Contrary to existing beliefs and practices, the lack of resources in low- and middle-income countries paradoxically increases the need for reliable healthcare evidence to prioritize the use of these scarce resources [4].

Highly qualified personnel are needed to propose, initiate and implement clinical trials. Such human resource development requires relatively stable, well-resourced research and higher education institutes, and well established science governance systems, which is not the case in developing countries [62]. Medical schools and teaching hospitals in LMIC have poorly prepared their graduates to conduct scientific trials and clinical research. In India for example, though there are half a million physicians with 50–60 physicians per 100,000 people, fewer than 200 have been trained in Good Clinical Practice (GCP) [55]. Moreover, mobility of highly skilled human resources due to the growing internationalization of the labour market is creating a permanent brain drain [62]. Other studies also reported numbers of qualified researchers not reaching critical mass, inadequate research infrastructure, and inconsistent and limited funding opportunities [64, 65] as factors that hampered the conduct of research in these countries. The Global Forum for Health Research report emphasized that strengthening research capacity in developing countries is one of the most effective and sustainable ways of advancing health and development in these countries and of helping correct the 10/90 gap in health research [61]. Building scientific capacity is much more than simple science and technology transfer from the developed world to the developing world. The key to scientific success resides in human resources. The emphasis must therefore be on training in an equitable, respectful way and on establishing long-lasting, sustainable partnerships [63].

Ethical and regulatory system obstacles emerged as the second most important barrier. Lengthy ethical and regulatory review time created delays in implementing grants and sometimes led grants to expire before recruitment started. Other studies also reported lengthy or ill-defined approval processes, significant bureaucracy, and lack of regulatory staff with expertise in reviewing [66, 67]. Ethics and regulatory review procedures are critical for protection of the safety and interests of the participants. However, complex and overly strict ethical and regulatory systems could worsen the negative feedback loop between limited research capacity and small numbers of trials conducted. European experience showed that over-management and over-regulation might negatively affect research and how important it is to harmonize and not overregulate the field of clinical research [68, 69]. To address these problems in the ethics and regulatory approval systems, some capacity strengthening activities have been initiated through grants from the developed world [70].

Moreover, an inadequate research environment and various operational barriers, including complex and lengthy financial and logistic systems, appeared in many studies. Challenges related to patient recruitment were also reported. These are all very important barriers for sponsors and researchers as they may directly influence the time and budget allocated to run trials. For example in Europe, because of the substantial increase in costs and administrative burdens for implementation, international collaboration in academia-driven clinical research has decreased [71].

In general, barriers for conducting clinical trials were similar between health professionals and researchers However, this review found a considerable variation regarding lack of time as a barrier between these two groups. For studies that involved health professionals, competing demands (particularly lack of time) appeared to be the second predominant theme. Lack of time was a less important barrier for clinical researchers. It is well known that most clinicians around the world have competing priorities that require them to engaging on caring for patients. For example, one systematic review regarding barriers for participation of doctors in clinical trials in developed countries identified lack of time as a major barrier [24]. Physicians in the developing world are already overstretched with responsibilities of patient care. However, the potential of scaling up clinical research in developing countries is unlikely to be attained without greater involvement of physicians. To keep a perfect balance between the clinical practice and research, it is proposed that the busy physicians should develop separate specialized teams for providing high-end clinical care as well as conducting quality research, wherein he/she plays the role of a leader to supervise and guide them [72]. Leadership commitment to practicing clinicians can also improve the degree of clinical-trial participation through supportive managerial functions, including time and space allocations and individual recognition [72, 73].

Exploiting the enormous research potential in developing countries has a double contribution as it can address some of the challenges that face the conduct of clinical trials in the developed world. Recruitment of trial participants is easier than in the developed world; large outcome trials that require enrolment of thousands of patients could make huge savings for the sponsor if the trial is conducted outside of developed countries [74]. Subject recruitment is responsible for around 23% of total trial costs, and 87% of US trials fail to meet temporal recruitment and enrolment milestones [75]. Moreover, the availability of large numbers of tertiary qualified workers and the relatively low salary base in these countries reduces the cost of running clinical trials. A recent review indicated that about one-third of 509 clinical trials sponsored by US-based companies from 1995 to 2005 were conducted outside the USA, many in poor and low-income countries [19]. One reason for outsourcing is that international clinical trials often cost less than they do in the US.

While outsourcing and globalization of clinical trials is good for LMIC, funding should also extend to promoting investigator driven research by the local researchers. Developing countries should encourage clinical trials that primarily benefit their local population.

To realize this, instituting a system for wider implementation of local investigator-initiated trials is warranted. These trials are more applicable to local populations because they build on local healthcare knowledge [76]. They are more demand-led and responsive to a country’s needs because they are driven by a local or national agenda [77]. Besides, they are more likely to influence policy and sustainably link research to action [78].

International collaboration both from the developed world as well as within the developing world is crucial to foster research development in less developed countries. For example, India is one of the world’s fastest-growing clinical research destinations. The number of registered international clinical trials that include India have increased by 30% each year for the past three consecutive years for many of the reasons outlined above [79, 80]. Learning from and adapting best practices at all levels (system, organizational and individual) could be beneficial. Establishing a national level support group is warranted to address the various aspects of challenges in conducting trials, by providing mentoring support for the entire trial process from grant procurement to final report writing, and to play an advocacy role in streamlining funding and regulatory processes [46].

This review has limitations and strengths. It is obvious that most of the developing countries were hardly represented in the literature. Barriers may vary widely depending on the context in which the clinical trials are conducted. There are several inter-country differences in culture, socioeconomic and political contexts, therefore our findings may not reflect the situation in any specific country. Because of the limited number of articles in the review, our analysis did not examine the similarities and differences between barriers among the different developing countries. Most of the included studies were qualitative studies with small sample size and narrow diversity of participants. We excluded papers which were not written in English. This is because the cost of translation was not feasible. However, we believe the rigorous compilation of stakeholder view and experiences lays the foundation to guide future studies.

## Conclusion

There are wide variations within developing countries with respect to barriers in clinical trial initiation and implementation in these regions. Similarly, concerns may be different for foreign led versus local investigator initiated trials. Therefore, further studies need be conducted and should include diverse views from the different developing countries and various stakeholders.

## Additional files

Additional file 1: PRISMA 2009 Checklist. (DOC 62 kb)

Additional file 2: **Table S1.** Assessment of study quality for quantitative studies. (DOCX 29 kb)

Additional file 3: **Table S2. **Assessment of study quality for qualitative studies. (DOCX 31 kb)

## Abbreviations

AMANET: African Malaria Network Trust Trials
EDCTP: European Developing Countries Clinical Partnership
GCP: Good Clinical Practice
HIC: High Income Countries
LMIC: Low and Middle Income Countries
NCDs: Non-Communicable Diseases
PRISMA: Preferred Reporting Items for Systematic Reviews and Meta-Analyses
UK: United Kingdom
US: United States
WHO: World Health Organization
