# Supplementary material for: Barriers for conducting clinical trials in developing countries- a systematic review
Source: Int J Equity Health. 2018 Mar 22;17:37. doi: 10.1186/s12939-018-0748-6 (PMC5863824; doi:10.1186/s12939-018-0748-6)
Supplement: Supplementary file 2 — Table S1. Assessment of study quality for quantitative studies. (DOCX 29 kb) [file 12939_2018_748_MOESM2_ESM.docx]

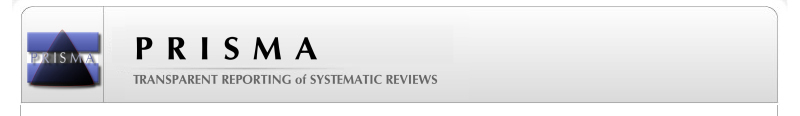
**PRISMA 2009 Flow Diagram**

**Screening**

**Included**

**Eligibility**

**Identification**

Records identified through database searching
(n = 3006)

Additional records identified through other sources
(n = 17)

Records after duplicates removed
(n = 1915)

Records screened
(n =1915)

Records excluded
(n = 1900)

Full-text articles assessed for eligibility
(n =15)

Articles are included in the analysis (n=15)
