# Supplementary material for: Barriers for conducting clinical trials in developing countries- a systematic review
Source: Int J Equity Health. 2018 Mar 22;17:37. doi: 10.1186/s12939-018-0748-6 (PMC5863824; doi:10.1186/s12939-018-0748-6)
Supplement: Supplementary file 3 — Table S2. Assessment of study quality for qualitative studies. (DOCX 31 kb) [file 12939_2018_748_MOESM3_ESM.docx]

| **Comment** | | **Response** | **Page number** |
| --- | --- | --- | --- |
| We thank Reviewer 1 for their constructive comments on our manuscript. Our responses to specific comments are outlined below. | | | |
| 1 | Please give the definition of "Clinical Trials" and "Developing Countries" in your study. | We have included definitions in the introduction section of the revised version of the manuscript, at lines 15-16 page 3 for developing countries and lines 26-28 page 3 for clinical trials. | 3 |
| 2 | Why did you choose the search date between 1995 and October 2015? | The review period (1995-2015) is chosen for the following reasons. Though an urgent call for country-specific health research to meet health needs of developing countries was made earlier (in 1990), guideline for conducting clinical trials only became available in 1995, effectively ushering in the era of modern clinical trials. This seemed to be an appropriate time to commence the review. In 2015, Harvard University researchers published a review paper and concluded that despite the overall increase in clinical trials over the last two decades, the progress of clinical trials in developing countries was particularly slow and challenging.  To describe this, we have added some text in the methods section (see italicised text below) on page 5, line 1-12:  *The Commission on Health Research for Development is an international initiative to study the status, effect, and needs of research on the health problems of developing countries. In the early 1990’s, the commission stated that the rapid expansion of country-specific health research to meet health needs of these countries was a necessity to encourage better health status for more people in settings with sometimes severely limited resources*[*1*](#_ENREF_1)*. In 1995, WHO published Guidelines for Good Clinical Practice*[*2*](#_ENREF_2)*, the world’s first standard for reporting clinical trials. We selected this as a logical starting point for assessing clinical trials from the modern era. In 2015, a review findings regarding the distribution of global clinical trials was released.*[*3*](#_ENREF_3) *This report concluded that despite the overall increase in clinical trials over the last two decades, the progress of clinical trials in developing countries was particularly slow and challenging. This milestone was accepted as a good point to conclude the search.* | 5 |
| 3 | Please provide the search strategy in one database, such as MEDLINE. | Thank you. we have included the PubMed search strategy in page 5, under the section ‘search strategy’ | 5 |
| 4 | In line 20 page 5, please provide the initials of researchers' name who screened the title and abstract. In line 56 page 5, please provide the initials of a third reviewer. | We have provided initials of all reviewers are provided in the revised version. | 5 |
| 5 | What is the eligibility criteria for title and abstract screening? The same with full text screening? | Yes, we used the same criteria for title and abstract screening. |  |
| 6 | In line 7-8, page 6, you mentioned that "Records of 20% of studies were compared between reviewers to ensure accuracy of data extraction". Which method did you use to compare? What is the comparison results? | Yes, we agreed with the reviewer’s comment–due to initial feasibility issues, only 20% of the records were compared. However, we went back and checked all papers when we had more resources-and so the 20% sentence has been removed.  To clarify this process, the data extraction and synthesis section is described in detail by adding additional paragraphs. The paragraphs briefly describe the methods and process of data synthesis, including the independent and group synthesis approaches to ensure sufficient and appropriate themes and subthemes are developed-see page 6, line 24-44. | 6 |
| 7 | The participants included in the 10 studies include many stakeholders, such as researchers, health professional, regulators, etc. It may be clearer if you could summarize the five themes by stakeholders, because different stakeholder may have different perceptions about clinical trials. | Thank you for this suggestion. In response, we have added a paragraph which summarises key findings by stakeholders on page 8-9 (starting from the last paragraph of page 8).  Two major stakeholders were identified from the included studies: researchers and health professionals. Both groups found similar barriers to conducting clinical trials However, this review found a divergence of views regarding lack of time as a barrier between these two groups. For studies that involved health professionals, competing demands (particularly lack of time) was the second most predominant theme, next to financial and human barriers.  Physicians in the developing world are overloaded with the responsibilities of patient care. However, the potential of scaling up clinical research in developing countries is unlikely to be attained without greater involvement of physicians. Therefore, we have discussed these issues in more detail and provide recommendations that might help busy clinicians to engage in research (see discussion section, page 10-11 (starting from the last paragraph of page 10). We believe that these details are appropriate and useful for the reader. | 8-9 and 10-11 |
| 8 | It is better if you can include your quality assessment results in the supplementary tables. | We have added two supplementary tables regarding quality assessment results, one for the quantitative and another one for the qualitative studies-on page 20. | 20 |
| 9 | The language writing and grammar need to be edited. | Done |  |
| We thank Reviewer 2 for their feedback on our manuscript. Our responses to Reviewer 2’s specific comments are outlined below. | | | |
| 10 | There were a few inconsistencies with formatting throughout, including: different font for reference numbers in-text, line 56; additional spaces, lines 58,59; missing full stop P4 L10; | Thank you. These has been checked and corrected. |  |
| 11 | Additionally, there were instances when writing could have been improved: P4, line 1 - 'conducting' rather than 'conduct' (this sentence is also very long); the objective P4 L27-28 could have been more concise; point one of eligibility criteria was clunky; P5 L34, missing 'of' (ascertainment the); P5 L53 - did you mean 'qualitative' rather than 'quality'; P9 L2-3, unclear | These has been checked and corrected. |  |
| 12 | Given the Newcastle-Ottawa scale was used to assess the quality of the quantitative studies, I wonder if using a checklist for studies that employed a qualitative methodology would have been useful (i.e. the COREQ checklist?). It would be useful to assess the rigour of the studies (if this was done - I would be explicit and include it in the criteria listed). It would also have been beneficial to see how quant studies scored. | Thank you for this comment. We did use a published method for assessing qualitative studies devised by Kuyper. We have added more detail on the quality assessment checklist for the qualitative studies on page 6, line 4-9. We have also added two supplementary tables that describe results of quality assessments both for the quantitative and qualitative studies (see page 20). | 6, 20 |
| 13 | It is unclear why 20% of records were compared between reviewers? | This point is covered by our response to the preceding question, where we described the approaches for data extraction and synthesis. | 6 |
| 14 | Perhaps it would have been more appropriate to undertake a meta-synthesis for the qualitative studies? It was unclear how the themes were determined (were they predetermined by reviewers, or in the results section of papers reviewed?). The methods section would benefit from some refinement. | Agree – the data extraction and synthesis section has been expanded and refined (page 6).  In brief, the evidence was synthesized using thematic analysis and synthesis[^4^](#_ENREF_4), where important or recurrent themes were identified by tabulating key concepts across studies. To avoid potential restrictions (from using predetermined themes) and to allow the possibility of including emergent key concepts, we used the study findings themselves to conduct a thematic analysis.  As the reviewer suggested, meta-syntheses offer novel interpretations of findings from qualitative studies[*^5^*](#_ENREF_5)*.* However*,* given that few studies are included-and due to the widely diverse settings as well as variations in methodological approaches across studies (which are important considerations in developing a more generalized mode[^5^](#_ENREF_5)^,^[^6^](#_ENREF_6), we did not conduct a meta-synthesis. | 6 |
| 15 | Why were articles not published in English excluded? | The review team can’t read other languages and the cost of translation was not feasible. We have clarified this in the limitation section on page 12. | 12 |
| 16 | It is an important area of research highlighting the need to increase efforts to take in to account contextual differences when implementing clinical trials. | Thank you! |  |

**References**

1. Evans JR. Essential national health research: a key to equity in development. Mass Medical Soc; 1990.

2. WHO Expert Committee. Guidelines for good clinical practice (GCP) for trials on pharmaceutical products, the use of essential drugs. Sixth report of the WHO Expert Committee, WHO Technical Report Series 1995.

3. Li R, Barnes M, Aldinger C, Bierer B. Global clinical trials: Ethics, harmonization and commitments to transparency. GLOBAL HEALTH 2015.

4. Thomas J, Harden A. Methods for the thematic synthesis of qualitative research in systematic reviews. BMC medical research methodology 2008;8:45.

5. Thorne S, Jensen L, Kearney MH, Noblit G, Sandelowski M. Qualitative metasynthesis: reflections on methodological orientation and ideological agenda. Qualitative health research 2004;14:1342-65.

6. Britten N, Campbell R, Pope C, Donovan J, Morgan M, Pill R. Using meta ethnography to synthesise qualitative research: a worked example. Journal of health services research & policy 2002;7: 209-15.
